# Supplementary material for: Development and Evaluation of Non-Antibiotic Growth Promoters for Food Animals
Source: Vet Sci. 2024 Dec 21;11(12):672. doi: 10.3390/vetsci11120672 (PMC11680322; doi:10.3390/vetsci11120672)
Supplement: Supplementary file 1 [file vetsci-11-00672-s001.zip › vetsci-3304507-supplementary.pdf]

veterinary sciences

MDPI

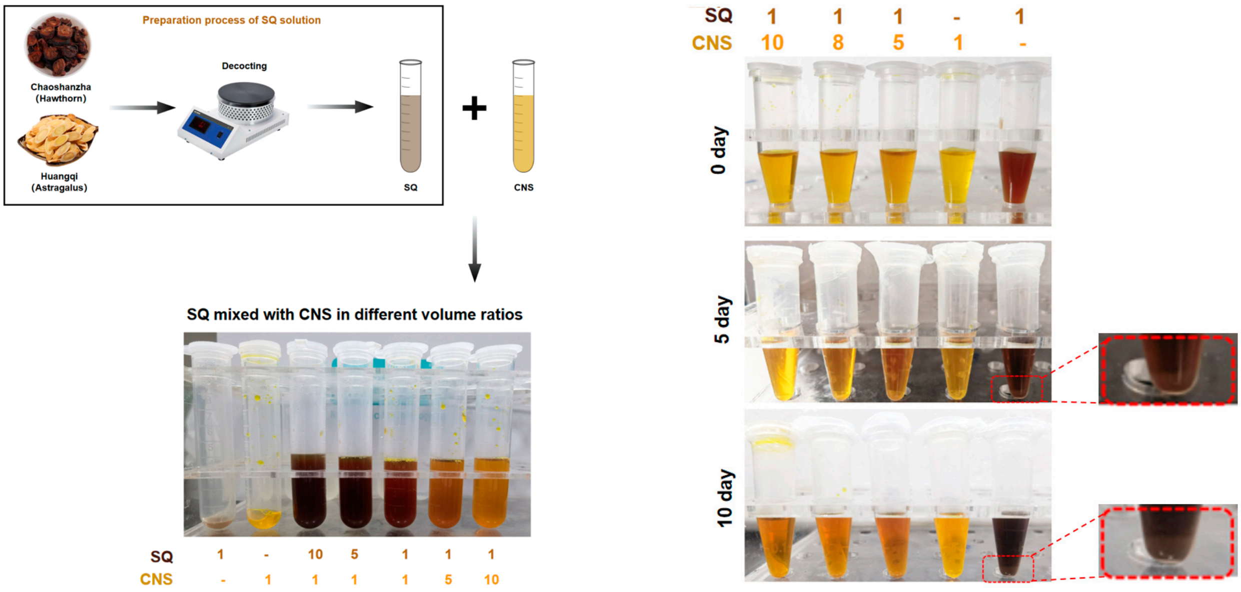

(**a**) (**b**)

Figure S1. Stability of compound super energy essence (**a**) Mutual solubility of SQ and CNS at different volume ratios; (**b**) Thermal stability of CSEE. The dashed red rectangles mark the precipitate.

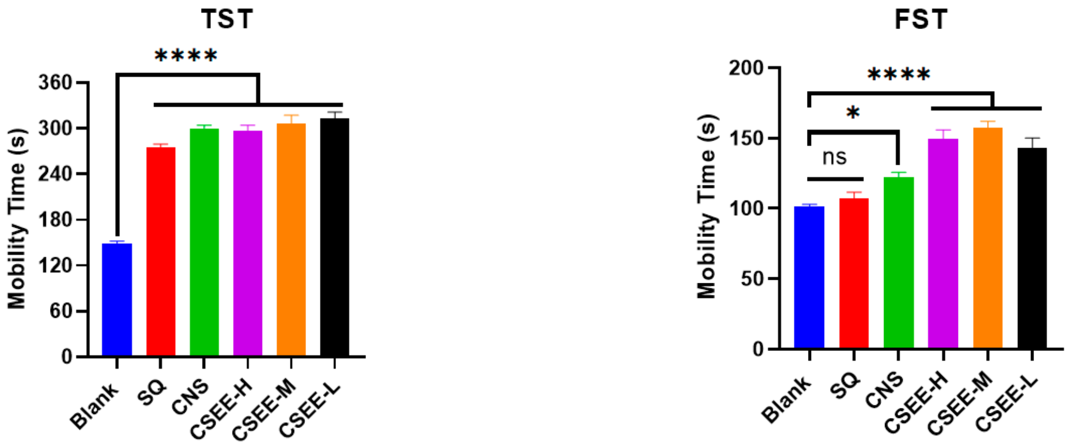

(**a**) (**b**)

Figure S2. Exercise times of mice in tail suspension test and forced swimming test (**a**) Exercise times of mice in tail suspension test and (**b**) forced swimming test. blank, blank control; SQ, SQ group (orally administered at a dose of 3 g/kg); CNS, CNS group; CSEE-H, SQ: CNS = 1:5 (*v*/*v*); CSEE-M, SQ: CNS=1:8 (*v*/*v*); CSEE-L, SQ:CNS=1:10 (v/v). n = 10. Analysis via one-way ANOVA, ****: $p < 0.0001$; *: $p < 0.05$; ns: not significant. Abreviations: FST， forced swim test； TST， tail suspension test.

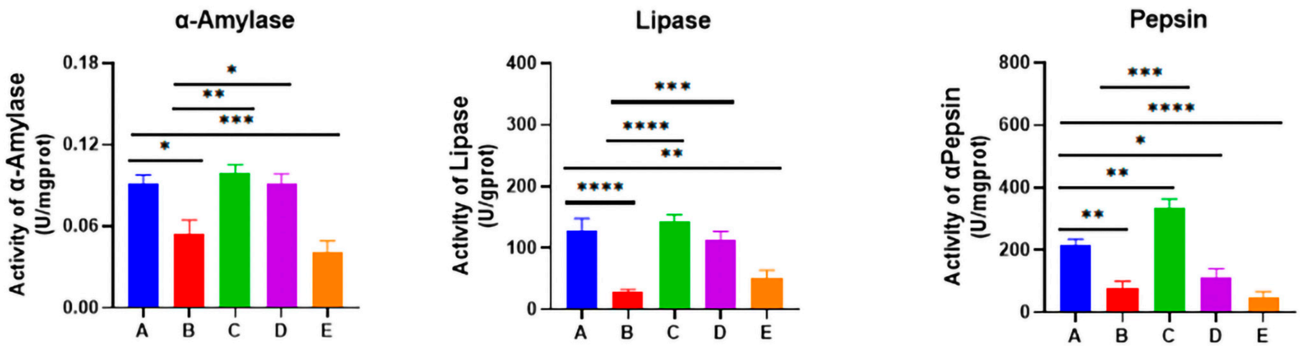

(**a**) (**b**) (**c**)

Figure S3. Digestive enzyme activity in the duodenum of broiler chickens (**a**) α-amylase activity; (**b**) Lipase activity; (**c**) Protease activity. Five broilers were slaughtered per group. A, blank group; B, guanidinoacetic acid group; C, 0.5% CSEE-M group; D, 1% CSEE-M group; E, 2% CSEE-M group. n = 10. Analysis via one-way ANOVA, *: $p < 0.05$; **: $p < 0.01$; ***: $p < 0.001$; ****: $p < 0.0001$.

Table S1. Content of CNS components per 1 kg.

Component	Content
Glycine	≥5000 mg/kg
L-Lysine	≥5000 mg/kg
DL-Methionine	≥5000 mg/kg
L-Arginine	≥1500 mg/kg
Vitamin A	300000~10 million IU/kg
Vitamin B_1	≥1000 mg/kg
Vitamin B_2	≥1000 mg/kg
Vitamin B_6	≥1500 mg/kg
Vitamin D_3	1~6 million IU/kg
Vitamin K_3	≥300 mg/kg
DL-α-tocopheryl acetate	≥5000 mg/kg
D-Biotin	≥100 mg/kg

Table S2. Primers sequences for qPCR.

Gene	Type	Primer sequences (5′ to 3′)
MyoD	Forward	CCTGCCCTCCACATCCTTTT
	Reverse	TCGCCCGCTTGAGGAATAAA
	Reverse	GGTCAGGGCACTCATGTCTC
Myf5	Forward	CTCAGGAATGCCATCCGCTA
	Reverse	CGTGATCCGATCCACAATGC
MSTN	Forward	CCCCCTCACGGTCGATTTT

	Reverse	GCGCTTACTTTGTGCTGTCC
IL-1β	Forward	GTGGGTACTGGAGAGTGGTCA
	Reverse	TGCATCTGTAGGAGTCCCTGT
GAPDH	Forward	ATCACTGCCACCCAGAAGAC
	Reverse	TGCCAGTGAGCTTCCCGTTC
β-actin	Forward	CTACCTCATGAAGATCCTGACC
	Reverse	GGCTGTATTCCCCTCCATCG

Table S3. Basic Feed Formulation.

**Trade Name**	**Pre-Broiler Feed 110**	**Medium Broiler Feed 811**
Brand	Huanggang Dongfang Hope Animal Nutrition Food Co Ltd	Zhengda Group
Food stage	0-21 days old broilers	≥21 days old broilers
Main raw material composition	Corn, soybean meal, puffed soybeans, corn protein meal, stone meal, DL-methionine, L-lysine sulphate, vitamins, sodium chloride, etc.	Corn, wheat, soybean meal, bran, rice bran, stone meal, sodium chloride, amino acids, amino acid salts, microorganisms and mineral elements, etc.
Ingredient list	Crude protein: ≥21.0%<br>Calcium: 0.5-1.8%<br>Ash: ≤12.0%<br>Total phosphorus: ≥0.40%<br>Crude fibre: ≤8.0%<br>Sodium chloride: 0.2-0.9%<br>Methionine: ≥0.40%<br>Moisture: ≤14.0%	Total protein: ≥19.0%<br>Calcium: 0.7-1.4%<br>Crude ash: ≤7.0%<br>Total phosphorus: ≥0.50%<br>Crude fibre: ≤6.0%<br>Sodium chloride: 0.3-0.8%<br>Methionine: 0.30-0.90%<br>Water content: ≤10.0%
Executive standard	GB/T 18823	GB/T 18823

Table S4. Unique microbiota and phenotypes of each group.

**Groups**	**Blank group**	**PC group**	**Treat group**	**Blank group & Treat group**	**Blank group & PC group**	**PC group & Treat group**
Unique microbiota	——	*Romboutsia*	*Helicobacter* and *Achromobacter*	*Lactobacillus* and *Lactobacillus aviarius*	——	*Streptococcus*
Unique phenotype	——	down-regulation of digestive enzyme activity; Fastest increase in body weight	Increase in survival rate;Increase in muscle content;Up-regulation of protease activity	Higher digestive enzyme activity	——	increased body weight
